# Tobacco Smoking and Second-Hand Smoke Exposure Impact on Tuberculosis in Children

**DOI:** 10.3390/jcm11072000

**Published:** 2022-04-02

**Authors:** Neus Altet, Irene Latorre, María Ángeles Jiménez-Fuentes, Antoni Soriano-Arandes, Raquel Villar-Hernández, Celia Milà, Pablo Rodríguez-Fernández, Beatriz Muriel-Moreno, Patricia Comella-del-Barrio, Pere Godoy, Joan-Pau Millet, Maria Luiza de Souza-Galvão, Carlos A. Jiménez-Ruiz, Jose Domínguez, on behalf of PII Smoking SEPAR Working Group

**Affiliations:** 1 Unitat de Tuberculosi Vall d’Hebron-Drassanes, Hospital Universitari Vall d’Hebron, 08001 Barcelona, Spain; mneusagomez@gmail.com (N.A.); m.jimenez@vhebron.net (M.Á.J.-F.); asoriano@vhebron.net (A.S.-A.); cmila@vhebron.net (C.M.); msouza@vhebron.net (M.L.d.S.-G.); 2 Serveis Clínics, Unitat Clínica de Tractament Directament Observat de la Tuberculosi, 08022 Barcelona, Spain; jmillet@aspb.cat; 3 Institut d’Investigació Germans Trias i Pujol, CIBER Enfermedades Respiratorias, Universitat Autònoma de Barcelona, 08916 Badalona, Spain; rvillar@igtp.cat (R.V.-H.); pablobena20@outlook.es (P.R.-F.); bmoreno@igtp.cat (B.M.-M.); patricia.comella@gmail.com (P.C.-d.-B.); jadominguez@igtp.cat (J.D.); 4 Institut de Biotecnologia i Biomedicina, 08193 Cerdanyola del Vallès, Spain; 5 Departament de Salut, Generalitat de Catalunya, 08028 Barcelona, Spain; pere.godoy@gencat.cat; 6 CIBER Epidemiología y Salud Pública, 28029 Madrid, Spain; 7 IRB-Lleida, Universitat de Lleida, 25198 Lleida, Spain; 8 Unidad de Tabaquismo de la Comunidad Autónoma de Madrid, 28015 Madrid, Spain; victorina@ctv.es

**Keywords:** *Mycobacterium tuberculosis*, cigarette smoking, passive smoking, child, immunology

## Abstract

Little is known about whether second-hand smoke (SHS) exposure affects tuberculosis (TB). Here, we investigate the association of cigarette smoke exposure with active TB and latent TB infection (LTBI) in children, analyzing Interferon-Gamma Release Assays’ (IGRAs) performance and cytokine immune responses. A total of 616 children from contact-tracing studies were included and classified regarding their smoking habits [unexposed, SHS, or smokers]. Risk factors for positive IGRAs, LTBI, and active TB were defined. GM-CSF, IFN-γ, IL-2, IL-5, IL-10, IL-13, IL-22, IL-17, TNF-α, IL-1RA and IP-10 cytokines were detected in a subgroup of patients. Being SHS exposed was associated with a positive IGRA [aOR (95% CI): 8.7 (5.9–12.8)] and was a main factor related with LTBI [aOR (95% CI): 7.57 (4.79–11.94)] and active TB [aOR (95% CI): 3.40 (1.45–7.98)]. Moreover, IGRAs’ sensitivity was reduced in active TB patients exposed to tobacco. IL-22, GM-CSF, IL-5, TNF-α, IP-10, and IL-13 were less secreted in LTBI children exposed to SHS. In conclusion, SHS is associated with LTBI and active TB in children. In addition, false-negative IGRAs obtained on active TB patients exposed to SHS, together with the decrease of specific cytokines released, suggest that tobacco may alter the immune response.

## 1. Introduction

Active smoking and second-hand smoke (SHS) exposure is a public health problem associated with active tuberculosis (TB) and latent TB infection (LTBI), as well as disease relapse and mortality [1]. In 2020, there were an estimated 9.9 million new TB cases worldwide, of which 11% were children (aged < 15 years). In addition, about 1.7 billion people (23% of the world’s population) are estimated to have LTBI, being at risk of disease progression during life [2]. Tobacco kills around 8 million people/year across the world. From these deaths, around 1.2 million are due to SHS exposure in non-smokers [3,4]. Infants are especially susceptible to SHS exposure, being vulnerable to developing health problems and having a significant risk factor for TB progression [3]. A systematic review conducted by the WHO and the International Union Against Tuberculosis and Lung Diseases indicated that the association of LTBI and active TB with tobacco smoke (passive or active) was independent of alcohol abuse, socioeconomic status, and other variable confounders. Furthermore, it is estimated that smoking can increase the risk for TB disease more than two-and-a-half times [1].

Even though there is an absence of a gold standard test for LTBI diagnosis, Interferon (IFN)-gamma (γ) Release Assays (IGRAs) are more specific than tuberculin skin test (TST) for this purpose because they do not present a cross-reaction with the BCG vaccine or non-tuberculous mycobacteria. However, their performance in children can be differentially affected by age, especially in those aged < 5 years [5]. Therefore, gaining evidence in this field using IGRAs is required, especially in children who are exposed to SHS [6,7,8,9]. Tobacco can increase the risk of infections by impairing immune defense mechanisms. Smoking promotes an inflammatory state leading to oxidative stress, mucosal inflammation, and the production of pro-inflammatory cytokines [10,11]. Altogether, this inflammatory environment can lead to a negative effect on the production of specific cytokines that play a role against infections, such as those caused by *Mycobacterium tuberculosis*. It has been described that cigarette smoke decreases specific *M. tuberculosis* cytokines in vitro [12,13]. In addition, has been shown that smokers have a reduced number of cells secreting Th1 cytokines, and as a consequence, can be more susceptive to viral or mycobacterial infections [14]. Interestingly, tobacco can also alter the IFN-γ response detected by IGRAs, impairing their assay performance [15]. However, only a few studies have addressed this impact in individuals exposed to SHS [16]. Here, we investigate the influence of cigarette smoke exposure on children as a risk factor of LTBI and active TB disease, analyzing IGRAs’ diagnostic performance and assessing specific cytokine immune responses against infection.

## 2. Material and Methods

### 2.1. Study Design and Sample Collection

This is a prospective study performed on children (<15 years old) who attended Unitat de Tuberculosi Vall d’Hebron-Drassanes (Barcelona, Spain) between September 2013 and September 2015. Patients were recruited from contact-tracing studies with an adult TB index case. Blood (<11 mL) was drawn from each patient for performing T-SPOT.TB (Oxford Immunotec, Abingdon, UK) and QuantiFERON-TB Gold In-Tube (QFN-G-IT, Qiagen, Düsseldorf, Germany) assays. The blood was sent to Institut d’Investigació Germans Trias i Pujol (IGTP, Badalona, Spain) to be tested by IGRAs.

This study was approved by the Ethical Committee of the Institut d’Investigació en Atenció Primària (IDIAP) Jordi Gol (protocol code P13/46). Informed consent was signed by the children’s parents or legal guardians before blood sampling. A questionnaire was collected from all patients detailing demographic information and clinical data, as well as index case data (diagnostic delay, bacteriology, and smoking condition) and degree of contact with index case (living together, days exposed, and daily contact hours). Exclusion criteria were HIV-positive, immunosuppression or past TB treatment for all patients enrolled in this study.

### 2.2. Study Population and Contact-Tracing Study Procedure

**Figure 1:**
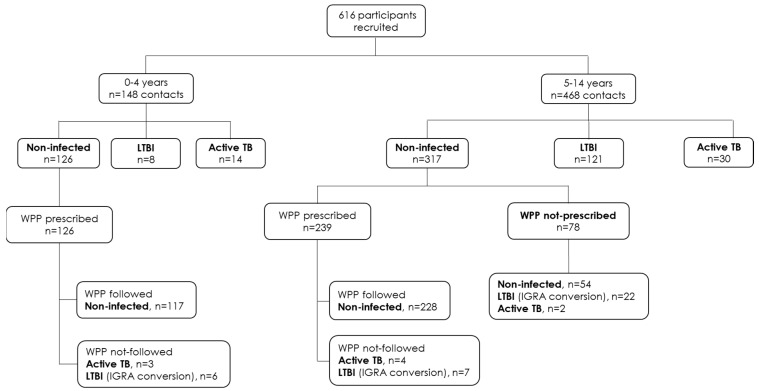
**Flow-chart with the final diagnosis of the 616 children included in the study.** Briefly, children coming from contact-tracing studies were stratified according to their age (<5 years old and 5–14 years old) and classified as uninfected, LTBI, and active TB. A Window Period Prophylaxis (WPP, primary prophylaxis) was indicated in the first screening in those children with negative TST and/or IGRAs. Then, after 8–12 weeks, a second screening was performed. In this second phase, TST and/or IGRAs were repeated. WPP was not prescribed in some cases due to non-acceptance.

A flow-chart detailing the contact-tracing study procedure and the final diagnosis of the children recruited is represented in Figure 1. Contact-tracing studies were conducted according to Spanish guidelines [17,18]. TST, IGRAs, and chest radiography were performed in the first visit during the contact-tracing study. If the radiography was abnormal, clinical and microbiological studies for active TB diagnosis were performed. In those exposed children with negative assays for tuberculosis diagnosis infection, a Window Period Prophylaxis (WPP, primary prophylaxis) was prescribed until a second phase of the study. In this second phase, an IGRA and/or TST (only in those cases with initial negative TST) were repeated 8–12 weeks after the initial visit. Radiological images and studies for active TB were conducted if needed. Patient groups were classified as: (i) LTBI contacts with an active TB case and normal chest radiograph. LTBI was diagnosed when a positive result was obtained with either of the two IGRAs (QFN-G-IT and/or T-SPOT.TB). (ii) Active TB cases with a *M. tuberculosis* positive culture (sputum bacteriology or gastric aspirate) or a probable TB based on clinical evaluation, compatible radiological images (when chest radiography was doubtful, a thoracic computerized tomography was performed), and clinical response to treatment. Having a positive TST/IGRA or being closely exposed to an active TB case was also used as diagnostic support. Lastly, (iii) uninfected individuals with negative TST and/or IGRA, and a normal chest radiograph imaging.

Children were classified regarding their smoking habits: (i) tobacco unexposed; (ii) SHS exposed; and (iii) smokers. Tobacco consumption was assessed by two independent interviews as described previously [6]. The pack-years ratio was calculated as (number of cigarettes consumed per day/20) × (number of years the person has smoked) [19]. SHS exposure was assessed in those children whose relatives currently smoked at home, estimating pack-years exposure for the year preceding examination of the child as a contact.

### 2.3. Tuberculin Skin Test, T-SPOT.TB and QFN-G-IT

TST was performed using 2 tuberculin units of PPD RT23 (Statens Serum Institut, Copenhagen, Denmark) and evaluated within 48–72 h by specialized nurses and physicians. Indurations ≥ 5 mm were considered positive according to the Spanish Pneumology and Thoracic Surgery Society guidelines [17,18]. T-SPOT.TB and QFN-G-IT were done and interpreted according to the manufacturer’s instructions.

### 2.4. Cytokine Detection by Means of a Bead-Based Multiplex Assay

Cytokine detection was performed on QFN-G-IT supernatants using a bead-based multiplex assay (Luminex 11-plex cytokine kit, R&D Systems, Minneapolis, MN, USA) and analyzed using Bioplex manager software (version 5.0, Bio-Rad, Hercules, CA, USA). Cytokines with a high relevance role on the immune response against *M. tuberculosis* were selected: granulocyte-macrophage colony-stimulating factor (GM-CSF), IFN-γ, interleukin (IL)-2, IL-5, IL-10, IL-13, IL-22, IL-17, tumour necrosis factor (TNF)-alpha (α), IL-1RA and IFN-γ-induced protein (IP)-10. The specific response was measured on antigen-stimulated plasmas after the subtraction of cytokine unstimulated concentration.

### 2.5. Statistical Methods

Comparisons between qualitative variables were done using the chi-square and Fisher’s exact tests. Risk factors for (i) positive IGRAs and for (ii) LTBI and active TB were defined using an adjusted Odds Ratio (aOR). Sensitivity, specificity, and predictive values (PVs) were calculated for TST, QFN-G-IT, and T-SPOT.TB. Data were analyzed using Epi Info 7.1.2 (www.cdc.gov/epiinfo/). Graphs were represented using GraphPad Prism version 4 (GraphPad Software, Inc., San Diego, CA, USA). Spot-forming cells (SFCs) counted in T-SPOT.TB were considered as an overall RD1 response (sum of SFCs in ESAT-6 and CFP-10). Differences in cytokine levels between groups were assessed using the two-tailed Mann–Whitney U-test for pairwise comparisons. Differences were considered statistically significant when *p*-values were <0.05.

## 3. Results

### 3.1. Participant’s Characteristics and Final Diagnosis

A total of 616 children from contact-tracing studies were included. A flow-chart indicating the final diagnosis of the children recruited is represented in Figure 1. Children were grouped according to their age (148 children aged < 5 years old and 468 children aged between 5–14 years old). LTBI and active TB were initially diagnosed in 8/148 (5.4%) and 14/148 (9.5%) of the children aged < 5; and in 121/468 (25.9%) and 30/468 (6.4%) of the children aged between 5–14 years, respectively. After the WPP, the total LTBI and active TB cases were 14/148 (9.5%) and 17/148 (11.5%) in children < 5 years; and 150/468 (32.1%) and 36/468 (7.7%) in 5–14 years children, respectively. Globally, active TB was higher in children < 5 years (11.5%) when compared with the group of children aged between 5–14 years old (7.7%), however, differences were not significant.

### 3.2. Risk Factors Associated with a Positive IGRA

**Figure 2:**
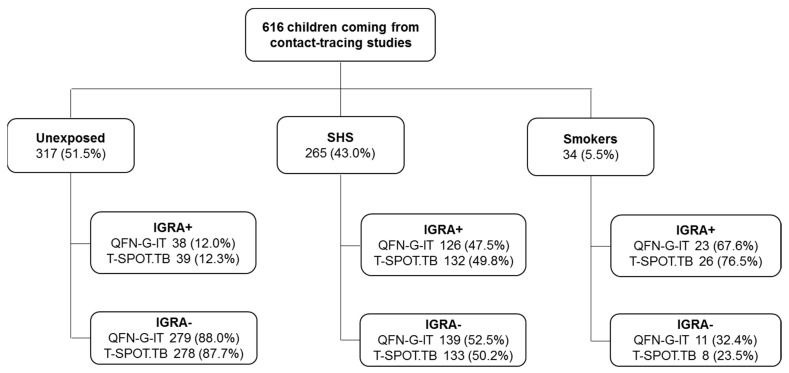
**IGRA positive or negative results stratification according to the smoking habit.** T-SPOT.TB and QFN-G-IT results in the 616 children recruited in the study, and stratification regarding their tobacco exposure (unexposed, SHS, or smokers).

IGRAs’ performance was globally assessed according to smoking habits, showing that the QFN-G-IT or T-SPOT.TB positivity rate increased in children exposed to SHS or smokers (for QFN-G-IT: 12.0% vs. 47.5% vs. 67.6% in unexposed, SHS and smokers respectively; for T-SPOT.TB: 12.3% vs. 49.8% vs. 76.5% in unexposed, SHS and smokers respectively). On the contrary, the highest negative percentages were observed in unexposed children (for QFN-G-IT: 88.0% vs. 52.5% vs. 32.4% in unexposed, SHS and smokers respectively; for T-SPOT.TB: 87.7% vs. 50.2% vs. 23.5% in unexposed, SHS and smokers respectively) (Figure 2).

Table 1 shows the main demographic characteristics and the possible risk factors associated with positive IGRAs (QFN-G-IT and/or T-SPOT.TB). Being exposed to SHS and/or being a smoker was significantly associated with having a positive IGRA [aOR and 95% confidence interval (CI): 8.7 (5.9–12.8) for SHS; and 25.6 (9.95–70.5) for smokers; *p* < 0.00001 for both conditions; Table 1]. This risk was also significantly higher when pack-years exposure increased [aOR (95% CI): 5.20 (3.4–8.0) for 1–15 pack-years exposure; and 23.8 (13.7–41.4) for >15 pack-years exposure; *p* < 0.00001]. Moreover, being a contact of a smoking index case was an important risk factor as well [aOR (95% CI): 2.47 (1.72–3.53); *p* < 0.00001].

### 3.3. Risk Factors for LTBI and Active TB

Being exposed to SHS and/or being a smoker was one of the main factors associated with LTBI, together with pack-years exposure to tobacco smoke. These factors were also associated with active TB, except for the smoker group, where no significant differences were observed. Risk factors associated with the latter group were difficult to establish due to the low number of children with active TB cataloged as smokers (*n* = 7). Acid-fast bacilli sputum grade of the index case, and having a daily contact of >6 h were other two risk factors associated with LTBI and active TB (Table 2).

### 3.4. Sensitivity Values for Active TB According to Tobacco Smoke Exposure

As shown in Table 3, IGRAs’ sensitivity decreased in children with active TB who were directly or indirectly exposed to tobacco smoke, being that the T-SPOT.TB is more sensitive than the QFN-G-IT. All these data may indicate that tobacco exposure can be associated with false-negative IGRA results when diagnosing active TB. The percentage of active TB cases was significantly higher in individuals exposed to SHS or smokers when compared with unexposed [3.1% (10/317) in unexposed children, vs. 13.6% (36/265; *p* < 0.000001) in SHS exposed, and vs. 20.6% (7/34; *p* < 0.001) in smokers]. Finally, TB disease prevalence increased based on tobacco smoke exposure, being higher in children exposed to SHS and/or being smokers [% disease prevalence (95% CI): 3.15 (1.52–5.72) in unexposed vs. 13.48 (9.63–18.17) in SHS exposed vs. 20.59 (8.70–37.9) in smokers]. Interestingly, the percentage of positive IGRAs’ results increased with tobacco exposure due to the high risk of infection. As a consequence, the specificity for diagnosing active TB in contact-tracing is reduced.

### 3.5. Cytokine Responses in Children Exposed to SHS

**Figure 3:**
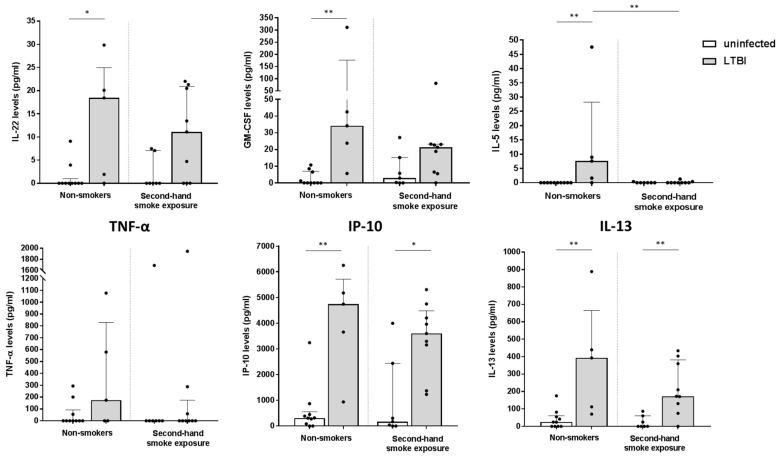
**Specific cytokine responses against *M. tuberculosis* regarding the smoking habit.** GM-CSF, IFN-γ, IL-2, IL-5, IL-10, IL-13, IL-22, IL-17, TNF-α, IL-1RA and IP-10 cytokine levels (pg/mL) were analysed in a subgroup of uninfected controls (unexposed *n* = 10 and SHS *n* = 7) and LTBI individuals (unexposed *n* = 5 and SHS *n* = 9). Values obtained from the negative control tube were subtracted from the antigen-specific tube. Bars depict medians with interquartile ranges. Differences between conditions were calculated using the two-tailed Mann–Whitney U-test. Only significant differences between comparisons are indicated in the graphs. * *p* < 0.05; and ** *p* < 0.01.

We investigated whether SHS could affect cytokine responses (other than IFN-γ) against *M. tuberculosis* infection. For this purpose, cytokine responses were analyzed in a subgroup of unexposed and SHS exposed patients, classified as uninfected controls or LTBI. Tobacco unexposed children had a significantly higher secretion of IL-22, GM-CSF, and IL-5 when infected than when they were not (*p* < 0.05 for IL-22; and *p* < 0.01 for both GM-CSF and IL-5). In contrast, LTBI children exposed to SHS failed to secrete significantly more IL-22, GM-CSF, and IL-5 than those exposed to SHS who were uninfected. In addition, IL-5 levels were significantly higher in tobacco unexposed LTBI compared to those LTBI individuals exposed to SHS (*p* < 0.01). Although differences for TNF-α in unexposed and SHS exposed children were not significant when comparing controls and infected individuals, the level of this cytokine tended to be reduced in children exposed to SHS [median (pg/mL) and interquartile range (IQR): 174.8 (0–828.5) for LTBI individuals unexposed to tobacco *versus* 0 (0–173.7) for LTBI individuals exposed to SHS]. For IP-10 and IL-13, cytokine responses were significantly higher in LTBI individuals than in uninfected controls for both conditions (unexposed: *p* < 0.01 for both IP-10 and IL-13; and SHS exposed: *p* < 0.05 for IP-10 and *p* < 0.01 for IL-13) (Figure 3). There were no significant differences between groups for IL-1RA, IL-10, and IFN-γ. A low production of IL-2 and IL-17 cytokines was observed (data not shown).

No reduction in IFN-γ production was associated with SHS exposure using the bead-based multiplex assay. These results are in agreement with those obtained by IGRAs (QFN-G-IT and T-SPOT.TB). On this matter, no significant differences were obtained on the IFN-γ released in QFN-G-IT or SFCs (overall RD1 response) counted in T-SPOT.TB between unexposed and individuals exposed to SHS with LTBI [median and IQR for IFN-γ released in QFN-G-IT (IU/mL): 2.29 (0.96–14.58) for unexposed and 3.55 (1.02–9.57) for SHS; for SFCs in T-SPOT.TB: 46.00 (20.00–94.00) for unexposed and 42.50 (21.00–82.50) for SHS].

## 4. Discussion

Tobacco affects lungs’ health, causing a negative impact on disease development such as cancer, chronic pulmonary diseases, or infectious diseases such as TB. In addition, there is no consensus about whether SHS exposure affects TB susceptibility. Here, we investigate the influence of direct tobacco smoke and SHS exposure as TB risk factors on children. Furthermore, the present study assesses the patient’s immune response by means of IGRAs and measures multiple cytokines involved in the response against the bacilli. Our results demonstrate that being exposed to SHS and/or a smoker was one of the main risk factors associated with LTBI and active TB. Interestingly, children with active TB disease who were smokers or exposed to SHS had a higher number of false-negative IGRAs, suggesting that tobacco may alter the *M. tuberculosis* mediated immune response favoring disease progression. Finally, cytokines such as IL-22, GM-CSF, IL-5, TNF-α, IP-10, and IL-13 were less secreted in children exposed to SHS with LTBI.

In a previous study from the group published in 2017 [6], we assessed tobacco smoke’s influence on TB symptoms, culture conversions, and immune response. The results from this investigation evidenced that (i) smoking in adults was a LTBI risk factor, (ii) adult active TB patients who smoked had high rates of false-negative IGRAs, and (iii) the IFN-γ immune response measured by IGRAs decreased in smokers. This weakened response against the bacteria was also associated with the pack-years increase. Continuing with this research line, the present study provides a step towards better understanding tobacco smoke impact, investigating the SHS influence on 616 children from TB contact-tracing studies. Interestingly, we found that SHS was also associated with LTBI and active TB in children aged < 5 and between 5–14 years old. Although SHS association with active TB has been suggested by others, more investigation in this direction is needed. One study, which followed up more than 15.000 never-smoking women, found that females with active TB were more likely to have been exposed to SHS than those without the disease [20]. Moreover, a meta-analysis performed by Patra J. et al. [21] concluded that children exposed to SHS had a 3-fold increased risk of having active TB; this risk was higher in children than in adults. In this direction, another study conducted by Adetifa I. et al. [22] found an increased risk of infection in children who were closely exposed to a TB smoking index case when compared to a non-smoking case. Finally, a recent study has also shown in 9810 schoolchildren in Mongolia that QFN positivity was associated with exposure to environmental tobacco smoke [23]. Our results confirm that being a contact of a smoking index case was an important risk factor of having a positive IGRA. This can be explained by the fact that tobacco use may increase the risk of disease transmission due to several reasons such as (i) increased coughing, (ii) more severe disease, and (iii) chronic coughing leading to a long time of disease transmission. Altogether it is important to note that our work observes a clear association between SHS exposure and risk for LTBI or active TB in children, with the results being in line with those observed in previous studies. Interestingly, our findings also state that this risk is associated with an increase in pack-years SHS exposure.

IGRAs sensitivity is compromised when performed in active TB, nevertheless, it is shown here that this sensitivity is even lower due to tobacco smoke exposure. These findings are in agreement with our previous work in smoking adults [6]. We also found that children exposed to SHS or direct smokers had an increased probability of having a negative IGRA in the presence of the disease. Therefore, IGRAs results may be interpreted carefully in individuals exposed to cigarette smoke, especially in children. Altogether, as a consequence of this compromised assay performance, it would also be interesting to speculate about modifying threshold cut-offs of IGRAs when performed in smokers or individuals exposed to SHS, as has been suggested in other scenarios such as immunosuppressed individuals [24].

Some in vitro studies have shown that cigarette smoke or e-cigarette vapor can reduce specific *M. tuberculosis* cytokines such as TNF-α [12,13,25]; however, the influence of SHS exposure on the immune response is still poorly understood. Here we found that LTBI children exposed to SHS secreted less IL-22, GM-CSF, IL-5, TNF-α, IP-10, and IL-13 cytokines. It is important to point out that these cytokines have an important role in mycobacterial infections, and their reduction may indicate in some way a weakened immune system. While the roles of TNF-α as an effector Th1 cytokine, and IP-10 as a chemokine involved in monocytes and Th1 cells trafficking into the inflammatory foci are well-known in TB [26,27,28]; other cytokines like IL-22, GM-CSF, IL-5, and IL-13 are less studied. IL-22 is produced by adaptive immune cells including Th1 and Th17 lymphocytes. Some investigations indicate a protective role of IL-22 in respiratory infections, however, its link with TB is still not completely understood [29]. GM-CSF is essential to confine bacterial growth in experimental models [30]. IL-5 and IL-13 are Th2-associated cytokines. Both are found in *M. tuberculosis*-infected children at higher levels than uninfected ones, having a modulatory role on *M. tuberculosis*-specific T-cell responses [31,32]. Altogether, these data could indicate that specific *M. tuberculosis* Th1/Th2/Th17 host immunology might be altered by SHS exposure. The balance between these specific responses is vital to achieving effective disease control, stimulating and modulating inflammation, as well as improving granuloma formation [16].

Despite these findings, the limitations should be addressed. First, SHS exposure can act in conjunction with other risk factors such as biomass fuel use, contact degree with an active TB case, genetic factors, other infections, and socioeconomic disadvantages which could have also an impact on the disease or cytokine immune response impairment. Finally, in our study, SHS exposure was assessed the year preceding the contact examination of the child. Although the data obtained here is robust, results could likely be affected by all the previous years of SHS exposure before the child’s contact with the index case.

The findings in this study can imply important public health considerations, especially in children exposed to SHS at home or countries with poor smoking restrictions and high TB incidence. Additionally, future research on the association of third-hand smoke or e-cigarette vapor exposure with lung diseases should also be carried out. In summary, our study has added value to research on SHS exposure and its association with LTBI and active TB risk in children. Interventions should be done to promote smoking cessation or eliminate indoor smoking, and as a consequence reduce LTBI or disease transmission in children who are exposed to tobacco smoke.

## Figures and Tables

**Table 1:** Main demographic characteristics and risk factors according to positive IGRA results (QFN-G-IT and/or T-SPOT.TB positive result) in contact-tracing studies of children included in the study.

	Positive QFN-G-IT and/or T-SPOT.TB								
Variables	0–4 Years: 148 Contacts (Positive IGRAs, *n* = 31)			5–14 Years: 468 Contacts (Positive IGRAs, *n* = 181)			0–14 Years: 616 Contacts (Positive IGRAs, *n* = 212)		
	*n* (%) *	OR (95% CI)	*p*-Value	*n* (%) *	OR (95% CI)	*p*-Value	*n* (%) *	OR (95% CI)	*p*-Value
**Gender**									
*Female*	18 (22.0)	1	NS	79 (32.9)	1	<0.001	97 (30.1)	1	<0.05
*Male*	13 (19.7)	0.87 (0.4–1.9)		102 (44.7)	1.65 (1.1–2.4)		115 (39.1)	1.49 (1.06–2.1)	
**BCG**									
*No*	23 (19.0)	1	NS	78 (33.8)	1	<0.05	101 (28.7)	1	<0.005
*Yes*	8 (29.6)	1.8 (0.7–4.6)		103 (43.5)	1.51 (1.0–2.2)		111 (42.0)	1.80 (1.3–2.5)	
**Immigrant**									
*No*	22 (18.3)	1	NS	77 (33.5)	1	<0.05	99 (28.3)	1	<0.0005
*Yes*	9 (32.1)	2.1 (0.8–5.3)		104 (43.7)	1.5 (1.1–2.2)		113 (42.5)	1.87 (1.33–2.62)	
**Smoking Habit**									
*Unexposed*	7 (8.3)	1		34 (14.6)	1		41 (12.9) 144 (54.3)	1	
*SHS*	24 (37.5)	6.6 (2.4–18.6)	<0.00005	120 (59.7)	8.7 (5.31–14.1)	<0.00001		8.7 (5.9–12.8)	<0.00001
*Smoker* ^†^	--	--	--	27 (79.4)	22.6 (8.5–62.3)	<0.00001	27 (79.4)	25.6 (9.95–70.5)	<0.00001
**Pack-years exposed**									
*None*	8 (9.4)	1		35 (14.8)	1		43 (13.4)	1	
1 *to* 15	10 (24.4)	3.1 (1.01–9.7)	<0.05	71 (50.3)	5.8 (3.6–9.56)	<0.00001	81 (44.5)	5.20 (3.4–8.0)	<0.00001
>15	13 (61.9)	13.9 (4.01–50.4)	<0.00001	75 (83.3)	28.8 (14.9–55.8)	<0.00001	88 (78.6)	23.8 (13.7–41.4)	<0.00001
**Underweight**									
*No*	28 (20.3)	1	NS	165 (38.8)	1	NS	193 (34.3)	1	NS
*Yes*	3 (30.0)	1.68 (1.4–6.92)		16 (37.2)	0.93 (0.5–1.78)		19 (35.8)	1.07 (0.6–1.93)	
**IC drug-resistant**									
*No*	30 (21.4)	1	NS	166 (38.4)	1	NS	196 (34.3)	1	NS
*Yes*	1 (12.5)	0.52 (0.06–4.4)		15 (41.7)	1.14 (0.6-2.3)		16 (36.4)	1.09 (0.6–2.07)	
**Living Together**									
*No*	7 (11.5)	1	<0.05	72 (26.5)	1	<0.00001	79 (23.7)	1	<0.00001
*Yes*	24 (27.6)	2.9 (1.2–7.35)		109 (55.6)	3.48 (2.4–5.14)		133 (47.0)	2.85 (2.02–4.02)	
**Days Exposed**									
<50	19 (16.8)	1	<0.05	115 (31.4)	1	<0.00001	134 (28.0)	1	<0.00001
≥50	12 (34.3)	2.6 (1.01–6.6)		66 (64.7)	5.83 (3.6–9.5)		78 (56.9)	3.4 (2.26–5.14)	
**Daily contact hours**									
<6	8 (11.4)	1	<0.01	74 (25.3)	1	<0.00001	59 (23.0)	1	<0.00001
≥6	23 (29.5)	3.24 (1.3–4.83)		107 (61.1)	4.7 (3.1–6.96)		153 (42.5)	2.47 (1.72–3.53)	
**IC Smoker**									
*No*	11 (14.1)	1	<0.05	48 (27.0)	1	<0.0001	59 (23.0)	1	<0.00001
*Yes*	20 (28.6)	2.44 (1.07–5.4)		133 (45.9)	2.29 (1.53–3.43)		153 (42.5)	2.47 (1.72–3.53)	
**IC Diagnostic Delay**									
<50 days	14 (17.7)	1	NS	75 (32.1)	1	<0.005	89 (28.4)	1	<0.005
≥50 days	17 (24.6)	1.52 (0.6–3.6)		106 (45.3)	1.76 (1.18–2.6)		123 (40.6)	1.72 (1.21–2.44)	
**IC AFB sputum grade**									
1 + (10–99/100 fields)	3 (5.9)	1		32 (23.9)	1		35 (18.9)	1	
2 + (1–10/field)	15 (20.3)	4.1 (1.05–23.0)	<0.05	95 (41.3)	2.24 (1.4–3.7)	<0.05	110 (36.2)	2.4 (1.5–3.8)	<0.0001
3 + (>10/field)	13 (56.5)	20.8 (4.3–127.2)	<0.00001	54 (40.3)	3.44 (2.0–6.0)	<0.00001	67 (52.3)	4.7 (2.8–7.8)	<0.00001

* Percentages are calculated over the total amount of the given variable. ^†^ No children < 5 years were classified as smokers in this study. OR: Odds Ratio; CI: Confidence Interval; SHS: Second-hand smoke; IC: index case; AFB: acid-fast bacilli; NS: not statistically significant.

**Table 2:** LTBI and active TB risk factors in children included in the study.

Variable	LTBI *		Active TB	
	aOR (95% CI)	*p*-Value	aOR (95% CI)	*p*-Value
**Gender (Male)**				
*No*	1	<0.05	1	NS
*Yes*	1.73 (1.13–2.64)		1.64 (0.81–3.32)	
BCG				
*No*	1	NS	1	NS
*Yes*	0.65 (0.15–2.79)		1.68 (0.06–47.10)	
**Age Group**				
0–4 years	1	<0.05	1	NS
5–15 years	2.59 (1.47–4.57)		0.46 (0.20–1.03)	
**Immigrant**				
*No*	1	NS	1	NS
*Yes*	2.25 (0.53–9.61)		0.42 (0.03–6.01)	
**Smoking Habit**				
*Unexposed*	1		1	
*SHS*	7.57 (4.79–11.94)	<0.00001	3.40 (1.45–7.98)	<0.005
*Smokers*	21.71 (8.18–57.60)	<0.00001	3.31 (0.80–13.76)	NS
**Pack-years exposed**				
*None*	1		1	
1 *to* 5	3.54 (1.94–6.44)	<0.00001	1.02 (0.20–5.19)	NS
6 *to* 15	10.30 (5.60–18.97)	<0.00001	3.52 (1.19–10.36)	<0.05
>15	18.21 (9.80- 41.81)	<0.00001	7.31 (2.61–20.49)	<0.0005
**Underweight**				
*No*	1	NS	1	<0.05
*Yes*	1.09 (0.52–2.29)		3.64 (1.30–10.14)	
**IC AFB sputum grade**				
1 + (10–99/100 fields)	1		1	
2 + (1–10/field)	2.83 (1.66–4.80)	<0.0001	7.49 (1.65–34.02)	<0.005
3 + (>10/field)	4.93 (2.61–9.33)	<0.00001	24.80 (5.31–115.5)	<0.00001
**Daily contact hours**				
<6	1	<0.00001	1	<0.00001
≥6	4.00 (2.46–6.51)		7.65 (3.17–18.5)	
**Days Exposed**				
≥50	1	NS	1	NS
<50	0.97 (0.55–1.71)		1.18 (0.53–2.65)	

* LTBI was defined as having positive IGRAs (T-SPOT.TB and/or QFN-G-IT) and chest radiography without alterations. OR: Odds Ratio; CI: Confidence Interval; SHS: Second-hand smoke; IC: index case; AFB: acid-fast bacilli; NS: not statistically significant.

**Table 3:** Sensitivity, specificity, and predictive values of TST, QFN-G-IT, and T-SPOT.TB according to direct and/or indirect tobacco smoke exposure in the 616 children recruited during contact-tracing studies.

Tobacco Smoke Exposition	Test	TB Cases	Non TB Cases	Sensitivity (95% CI)	Specificity (95% CI)	PPV % (95% CI)	NPV % (95% CI)
**Unexposed** ***n* = 317**	*TST* ≥ 5 mm	10	95	100	69.1	10	100
	*TST* < 5 mm	0	212	(69.1–100)	(63.6–74.2)	(8.2–11.1)	
***Disease Prevalence %*(95% CI)** 3.15 (1.52–5.72)	*QFN.G-IT pos*	10	28	100	90.9	26.3	100
	*QFN.G-IT neg*	0	279	(69.1–100)	(87.1–93.9)	(20.1–33.7)	
	*T-SPOT.TB pos*	10	29	100	90.5	25.6	100
	*T-SPOT.TB neg*	0	278	(69.1–100)	(87.2–94.3)	(12.6–39.7)	
**SHS** ***n* = 265**	*TST* ≥ 5 mm	36	136	100	40.6	21	100
	*TST* < 5 mm	0	93	(90.3–100)	(34.2–47.3)	(19.2–22.8)	
***Disease Prevalence %*(95% CI)** 13.48 (9.63–18.17)	*QFN.G-IT pos*	30	96	83.3	58.1	23.8	96
	*QFN.G-IT neg*	6	133	(67.2–93.6)	(51.4–64.5)	(20.2–27.8)	(91.4–97.9)
	*T-SPOT.TB pos*	31	101	86.1	55.9	23	96.2
	*T-SPOT.TB neg*	5	128	(74.8–97.4)	(49.0–62.4)	(20.1–27.2)	(91.8–98.3)
**Smokers** ***n* = 34**	*TST* ≥ 5 mm	7	23	100	14.8	23	100
	*TST* < 5 mm	0	4	(59.0–100)	(4.2–33.7)	(20.6–26.3)	
***Disease Prevalence %* (95% CI)** 20.59 (8.70–37.9)	*QFN.G-IT pos*	4	19	57.1	29.6	17.4	72.7
	*QFN.G-IT neg*	3	8	(18.4–90.1)	(13.7–50.2)	(9.6–29.5)	(48.7–88.2)
	*T-SPOT.TB pos*	7	19	100	29.6	26.9	100
	*T-SPOT.TB* *neg*	0	8	(59.0–100)	(13.7–50.2)	(22.4–32.0)	

SHS: Second-hand smoke; pos: Positive; neg: Negative; TB: tuberculosis; PPV: positive predictive value; NPV: negative predictive value; CI: Confidence Interval.

## Data Availability

Data contains potentially identifying/sensitive patient information and to publicly share data would breach patient confidentiality. However, excerpts of the data will be available upon request to the Research Ethical Committee of the Institut d’Investigació en Atenció Primària (IDIAP) Jordi Gol in Barcelona (contact email: pmoreno@idiapjgol.org), who will ensure that the data shared are in accordance with patient consent.
